# Characterization of a Salivirus (*Picornaviridae*) from a Diarrheal Child in Guatemala

**DOI:** 10.1128/genomeA.01751-15

**Published:** 2016-02-18

**Authors:** Terry Fei Fan Ng, Laura Magaña, Anna Montmayeur, Maria Renee Lopez, Nicole Gregoricus, M. Steven Oberste, Jan Vinjé, W. Allan Nix

**Affiliations:** a Division of Viral Diseases, Centers for Disease Control and Prevention, Atlanta, Georgia, USA; b Oak Ridge Institute for Science and Education, Oak Ridge, Tennessee, USA; c CSRA Inc., Falls Church, Virginia, USA; d Center for Health Studies, Universidad del Valle de Guatemala, Guatemala City, Guatemala

## Abstract

The complete genome sequence of a salivirus was identified in a stool sample from a Guatemalan child with acute gastroenteritis during a 2009 norovirus outbreak. This genome (genotype A1 strain GUT/2009/A-1746) shares 82% to 94% genome-wide nucleotide identity with saliviruses from the United States, China, Germany, and Nigeria, representing the first salivirus sequence from Central America.

## GENOME ANNOUNCEMENT

Picornaviruses are nonenveloped viruses with a single-stranded, linear, and positive-sense RNA genome that infect humans and other vertebrates (1). Salivirus is a new species of *Picornavirus* that has been detected in fecal samples from persons with diarrhea in the United States, Australia, Nigeria, China, Hong Kong, South Korea, and Germany (2–9), as well as in sewage (2, 10). Association with acute gastroenteritis has been proposed based on molecular and serological epidemiology studies (3, 4, 11).

In February 2009, a group of Guatemalan school children participating in a school excursion developed acute gastroenteritis (AGE) associated with GII.4 norovirus (12). Using a previously described viral metagenomics protocol (13, 14), we detected a divergent salivirus in one of the norovirus-positive samples. A near-complete salivirus genome was assembled using 34,983 reads generated by the Illumina MiSeq next-generation sequencing platform.

This salivirus sequence (SaliV-A strain GUT/2009/A-1746) was 7,952 bases long, with a G+C content of 56.8%. The genome organization is typical for saliviruses, with the configuration of 5ʹUTR^IRES-V^ (L-VP0-VP3-VP1-2A-2B-2C-3A-3B-3C^pro^-3D^pol^) 3ʹUTR-poly(A) (UTR, untranslated region; IRES, internal ribosome entry site).

There are 10 other salivirus complete or near-complete genomic sequences available in GenBank, seven with a complete polyprotein-coding region, and three with a near-complete polyprotein region. The available sequences vary from 6,366 to 7,978 nucleotides (nt) in length, excluding the poly(A) tail. The complete genome of the salivirus from Guatemala shares 94%, 91%, 91%, 90%, 89%, 82%, and 82% nucleotide identity with that of these other strains: NG-J1 (accession no. GQ179640, Nigeria/2007), 02394-01 (accession no. GQ184145, United States/2002), BN-2 (accession no. KP247439, Germany/2013), SH1 (accession no. GU245894, China/2009), CH (accession no. JN379039, chimpanzee/China/2011), BN-5 (accession no. KP247440, Germany/2013), and FHB (accession no. KM023140, China/2011), respectively. In the 828-nt VP1 capsid region, the Guatemalan virus shares 76% to 92% nucleotide identity to all salivirus VP1 nucleotide in GenBank (83% to 95% amino acid identity). The Guatemalan VP1 sequence shares the highest identity with strain NG-J1 (92% nucleotide and 95% amino acid identity) (8). This report confirmed the presence of salivirus in a child coinfected with norovirus in an acute gastroenteritis outbreak from Central America.

### Nucleotide sequence accession number.

The sequence of SaliV-A strain GUT/2009/A-1746 has been deposited in GenBank under accession no. KT310068.
